# Infrared Temperature Measurement Sensors of Overhead Power Conductors

**DOI:** 10.3390/s20247126

**Published:** 2020-12-12

**Authors:** Pablo Castro, Ramón Lecuna, Mario Manana, Maria Jose Martin, Dolores del Campo

**Affiliations:** 1 School of Mining and Energy Engineering, Electrical and Energy Engineering Department, University of Cantabria, Boulevard Ronda Rufino Peon lnº254, 39316 Torrelavega, Spain; ramon.lecuna@unican.es (R.L.); mananam@unican.es (M.M.); 2 Centro Español de Metrología (CEM), C/Alfar, nº2, 28760 Tres Cantos, Madrid, Spain; mjmartinh@cem.es (M.J.M.); ddelcampo@cem.es (D.d.C.)

**Keywords:** infrared thermometry, non-contact temperature measurement, ampacity, overhead power conductor, emissivity

## Abstract

Efficiency in power lines operation is becoming more crucial as the electrification increases and more renewable energies are connected into the grid. New methods and sensors are being added to create smart grids to face these challenges and conductor temperature sensors are one of them. Contact temperature sensors have several problems regarding safety and electronic damage due to the electromagnetic fields induced on the conductors. The goal of this paper is to describe an infrared temperature measurement sensor and to compare contact and non-contact temperature measurements to estimate the temperature of power lines. Measurements were done for almost a year, storing around 150,000 measures of contact and infrared thermometers for many different weather and load conditions. The results conclude that the infrared system can be successfully used to control the temperature of the overhead conductor within a range of less than 4 ${}^{\circ }$C difference with respect to contact temperature methods for the 88% of the samples and less than 6 ${}^{\circ }$C for the 99%.

## 1. Introduction

Temperature measurements of overhead high-voltage conductors are crucial to control some of the main parameters of the power lines, such as the sag, to control the minimum safety distance from the cable to the surroundings, and the ampacity, which is the maximum allowable current that the conductor can carry without damage [1,2,3].

With the development of smart grids, more and more data can be monitored, stored, and analyzed obtaining a better understanding of the electric grid. One of the methods improved with the increase of data acquisition is the dynamic line rating (DLR) [4], which measures the main weather and power line parameters to estimate the dynamic ampacity. Most of the methods to control these parameters use indirect measurements or contact temperature sensors [5,6,7]. Other methods and sensors for power line inspection are also reported [8,9,10].

Contact temperature measurements have several problems regarding safety and electronic damage due to the electromagnetic fields induced on the conductors. A non-contact temperature measurement method based on infrared thermometry is used in the temperature measurement comparison presented in this paper and it has been patented as Patent No. ES2542043A1 [11] and tested [12].

The two main challenges for the infrared temperature measurements of overhead conductors are to obtain a practical spot size and to estimate the emissivity of the material. A method to solve these questions is also explained in this paper.

## 2. Material and Methods

There are several ways to obtain an estimation of the temperature of an electric power cable. First, there are Standards provided by the two main international electric institutions, CIGRE (Conseil International des Grands Réseaux Électriques) [1] and IEEE (Institute of Electrical and Electronics Engineers) [2], which explain how to relate the current and the temperature based on the thermal balance of the conductor.

Second, computational fluid dynamic (CFD) and finite element methods (FEM) can also be used to study this thermal balance and to recreate different types of conductors and operation and weather conditions [13,14] and finally, direct or indirect measurement methods of some line parameters can be used to estimate the temperature of the conductor [15,16,17]. All the previous methods were used to check the reliability of the infrared thermometer measurements.

### 2.1. Standards Implementation

Both CIGRE and IEEE Standards describe the physics involved in the thermal balance of the conductor in steady and unsteady conditions, reaching similar results [18]. In this case study, a Matlab^®^ code was implemented based on the Equation (1) of CIGRE Standard to obtain the theoretical conductor temperature based on the weather and line operating conditions [19]. This algorithm was used to validate the numerical simulation.

(1) $$m\cdot c\cdot \frac{\Delta T}{\Delta t}=P_{j}+P_{m}+P_{s}-P_{c}-P_{r}$$

where:

- *m*: is the conductor mass per unit length
- *c*: is the conductor specific heat capacity
- ΔT: is the temperature increment
- Δt: is the time increment
- $P_{j}$: is the Joule heat gain
- $P_{m}$: is the magnetic heat gain
- $P_{s}$: is the solar heat gain
- $P_{c}$: is the convection heat loss
- $P_{r}$: is the radiation heat loss

### 2.2. Numerical Simulation

The main problems when trying to use infrared thermometers with power lines is the distance to the conductor and the unknown value of the conductor’s emissivity. On the one hand, there is a minimal safety distance between the conductor and the tower where the infrared thermometer would be placed. On the other hand, there is a maximum allowable distance for the infrared thermometer to have a good reading and a small spot size.

To increase the spot size and extend the possible distance between the thermometer and the conductor, a sphere was attached to the conductor. The sphere was simulated to check its influence on the conductor temperature and its cooling effect due to convection and radiation. The objective was to design a sphere of the same material as the external layers of the conductor (aluminum in this case) which was big enough for the spot size of the infrared thermometer but small enough to have controllable cooling effects. Additionally, attaching a specific manufactured sphere would solve the unknown emissivity problem.

Several diameters of the sphere were simulated and the cooling effect of the sphere acting as a fin was modelled. The domain included the conductor, with its iron core and aluminum layers, the aluminum sphere attached to the conductor, and the surrounding air volume. It was 1 m long and 0.2 m high and wide, with periodic boundary conditions at the front and the back of the conductor length to simulate an indefinitely long cable. The goals of the numerical model were the following:

- To recreate all the electrical and environmental conditions and obtain a realistic model of the behavior of the conductor and the sphere under operating conditions.
- To validate the results of the modelled conductor temperature with values obtained from the estimation of the temperature of the conductor under different conditions according to the Standards.
- To confirm and obtain a first approximation of the cooling effect of the sphere acting as a fin.

This last goal was important because the surface of the sphere in contact with the air increases the convection and radiation cooling. The physical phenomena simulated in the model were the following:

- The alternating current and the electromagnetic field associated with the conductor.
- Heat transfer effects resulting from conduction inside the cable and the sphere, natural and forced convection with the air surrounding both solids, solar heating, and cooling radiation to the atmosphere.

Therefore, the most important heat gain and loss mechanisms of the system were included in the numerical method. To include all these effects, the model was implemented by means of coupled physics solved by Ansys Workbench^®^. First the electromagnetic field was solved using Maxwell^®^ software and then the heat power $\left(W/m^{3}\right)$ generated inside the solids were translated into the computational fluid dynamic software Ansys Fluent^®^, where all the other physical and environmental conditions were recreated, and the temperature profile was calculated.

The model boundary conditions were the ambient temperature, the speed and direction of the wind, solar radiation and current. The main characteristics of the implemented numerical model were steady state analysis with gravity and variable air density, solar and surface to surface (S2S) radiation models, k-ϵ turbulence model, periodic conditions to simulate an indefinite long conductor and 890,784 elements. The thermo-physical properties of the conductor were the ones provided by the manufacturer and the value of the sphere emissivity was obtained from the estimation done by the Spanish Metrology Center (CEM).

**Figure 1:**
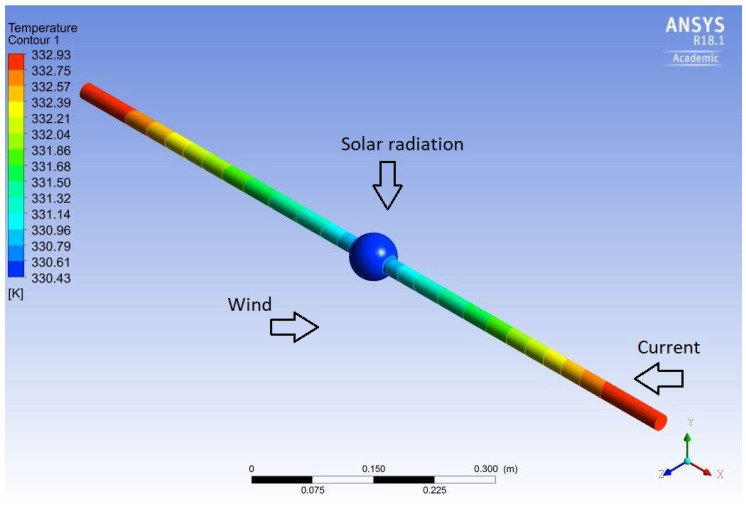
Boundary conditions and temperature profile of the conductor and the sphere.

**Figure 2:**
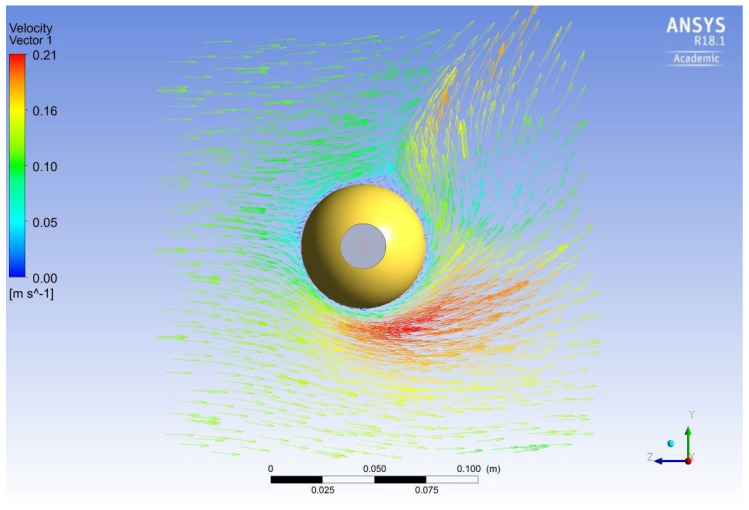
Wind velocity field around the sphere.

**Figure 3:**
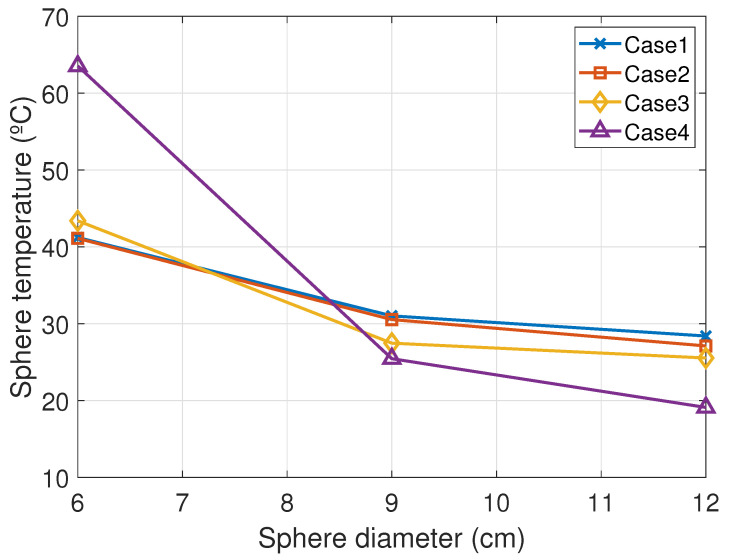
Simulated sphere temperature vs. sphere diameter.

Figure 1 presents the boundary conditions of the model and a temperature profile of the conductor and the sphere where the cooling effect of the sphere can be seen. Figure 2 shows an air velocity field surrounding the sphere resulting from the combined natural and forced convection. Three sphere diameters were simulated with different weather and line conditions and the results are summarized in Table 1. In Figure 3, the cooling effect of the sphere increasing with its diameter is clearly shown. The smallest simulated diameter was chosen to manufacture the sphere to minimize the cooling effect but to maintain a practical spot size for the pyrometer.

### 2.3. Infrared System Build Up

The two main components of the infrared system are the infrared thermometer and the sphere. To choose the thermometer, the criteria were available spot size, temperature range and cost. The minimum distance should be around 1 m, considering the length of the insulators typically attached to the tower and the spot size at that distance should not be bigger than 3 cm considering the size of the attached sphere.

Maximum allowable conductor temperature depends on the type of conductor but it is common to specify $50{\;}^{\circ }C$ or $70{\;}^{\circ }C$ as the maximum operational temperature so the needed temperature range would vary from a few degrees Celsius to around one hundred. The lower values of temperature measurements would not be an inconvenience because the maximum sag and ampacity values are fixed by the maximum allowable temperature, i.e., cold conductors are not the problem. Cost was also critical because this system should be a realistic alternative to the contact temperature instruments, which can be found for a few thousand euros.

The infrared thermometer chosen to fill these conditions was the commercial thermometer Optris CSLaser-SF50 model, with a temperature range between −30 ${}^{\circ }C$ and 1000 ${}^{\circ }C$, spectral range between 8 μm and 14 μm and an optical resolution of 50:1. The sphere had 60 mm of diameter with a hole of 21.8 mm of diameter and it was made of aluminum with a surface emissivity in the range of 0.95–0.98.

To obtain a value for the emissivity as accurate as possible, the sphere and two cables, one new and other used, were sent to the Spanish Metrology Center (CEM). The idea was to check the emissivity changes due to the conductor’s aging and to estimate the emissivity of the sphere which would be placed on the conductor. The measurement procedure was as follows:

Equipment

For infrared temperature measurements, a LAND C300 type pyrometer with the serial number 40002068 and the calibration certificate number 132525001 was used. The detection range of the pyrometer was 8–14 μm with a spot size of 4.5 mm diameter at 50 cm distance.

For contact temperature measurements, a Pt100 ISOTECH model with the serial number 191140/1 and the calibration certificate number 132564001 was used. It was connected to a thermometry bridge ASL F700 model with 100.0031Ω internal resistance with the serial number 005865/09 and the calibration certificate number 141034002.

Measurement Methods

To reach the different working temperatures, several methods were used:

- $5{\;}^{\circ }C$: The samples were introduced into a dewar with ice and waited until stabilization.
- $22{\;}^{\circ }C$: Laboratory controlled temperature was used $\left(\pm 1{\;}^{\circ }C\right)$.
- $50{\;}^{\circ }C$ and $80{\;}^{\circ }C$: A heating wire rolled over the samples was used.

**Figure 4:**
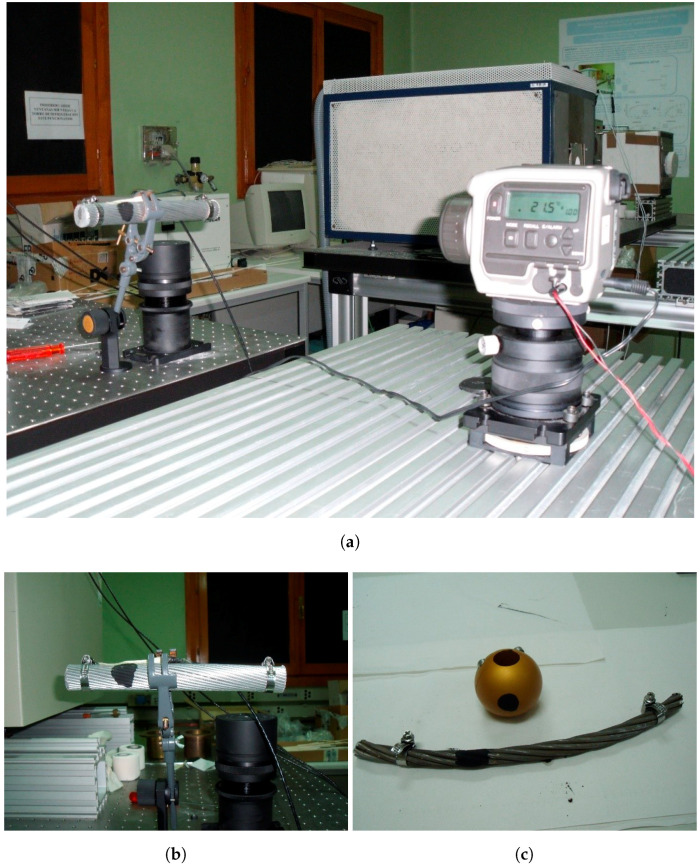
Samples with the high emissivity painting. (**a**) Infrared temperature measurements at CEM; (**b**) Samples with the high emissivity painting I; (**c**) Samples with the high emissivity painting II.

The samples were partially painted with a high emissivity painting (Nextel^®^). Contact temperature was measured with the calibrated Pt100 and the radiation temperature was measured with the pyrometer in the painted and the unpainted parts of the samples. Variations in the temperature measurements were used to obtain the values for the emissivity modifying its value from the pyrometer. Figure 4b,c show pictures of the samples with the high emissivity painting and Figure 4a shows a picture of the infrared temperature measurements. Temperature measurements were made at $t_{nominal}$ $5{\;}^{\circ }C$, $22{\;}^{\circ }C$, $50{\;}^{\circ }C$ and $80{\;}^{\circ }C$ for the new and used conductors and the sphere.

Contact temperature values from the Pt100, $t_{cont}$, and radiation temperature values from the pyrometer, $t_{rad}$, in painted and unpainted zones are summarized in Table 2. σ is the temperature stability during the measurements and *U* the calibration and measurement expanded uncertainty for 95% of probability. Columns 9, 10 and 11 of Table 2 indicate the differences between the contact temperature measurements and the radiation temperature measurements for ϵ = 1 (painted part) and ϵ<1 (unpainted part) and the difference between the two radiation temperature measurements.

**Figure 5:**
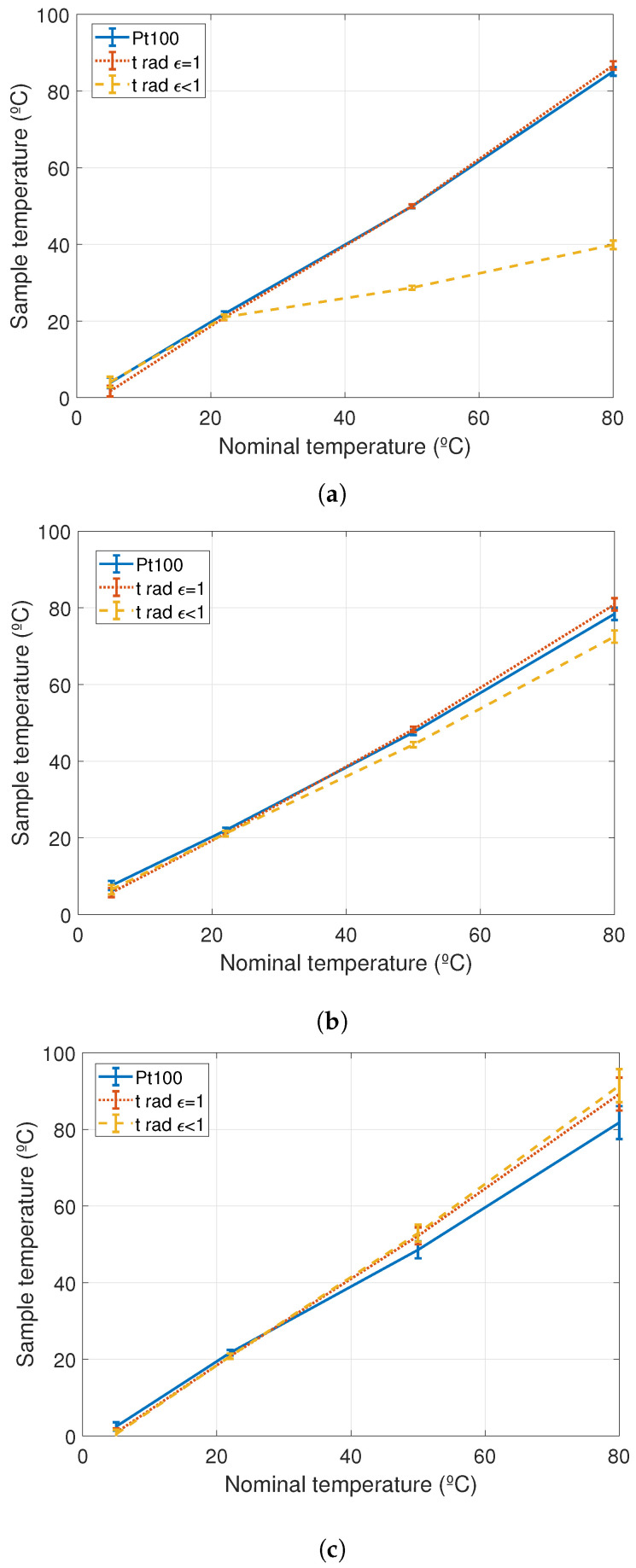
Contact and radiation temperature measurements of the samples. (**a**) Contact and radiation temperature measurements for the new conductor; (**b**) Contact and radiation temperature measurements for the used conductor; (**c**) Contact and radiation temperature measurements for the sphere.

Figure 5a–c show the contact and radiation temperature measurements for the 3 samples with their uncertainties. As can be seen in Figure 5a, the maximum temperature difference between the more accurate measurements (contact and infrared in painted zone) and the unpainted infrared temperature measurements corresponds to the new conductor.

Differences for used conductor and the sphere are less than $10{\;}^{\circ }C$ in the measured nominal temperature range but up to $45{\;}^{\circ }C$ in the case of the new conductor. This means that the emissivities for the old conductor and the sphere are close to 1 but for the new conductor is very low. New conductors have very shiny and polished surfaces with high reflectivity values which make the infrared temperature measurements more complicated. Aging and soiling of the conductor and the sphere contribute to increase the value of the emissivity making the estimations of infrared temperature measurements to improve with time if the emissivity values are corrected in the infrared thermometer.

Sample’s emissivity can be estimated using the pyrometer as the standard and changing the emissivity value of the equipment to reproduce the temperature differences obtained. In Table 3 the values of the estimated emissivities are summarized.

**Figure 6:**
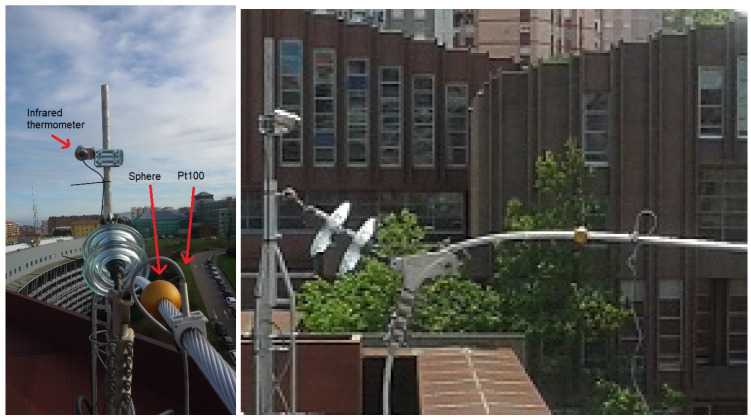
Pictures of the facility with the infrared thermometer and the Pt100.

Once the infrared thermometer and the sphere were characterized, the next step was to build a test facility to check the system behavior. An electric loop was installed in an outdoor facility to control the line parameters and measure the weather conditions with a weather station placed in site. The facility was performed with a high current and low voltage system to facilitate the operating conditions. Figure 6 shows two details of the facility.

**Figure 7:**
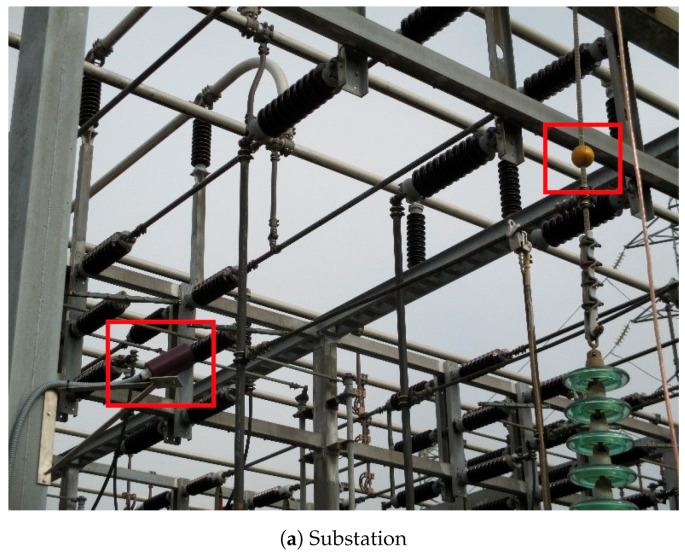

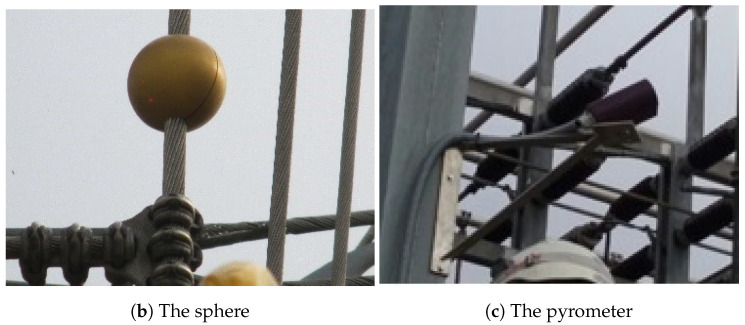
Pictures of the substation with the infrared thermometer and the sphere.

Two contact temperature sensors were placed, one close to the sphere and the other in the middle of the conductor, to check the temperature difference due to the cooling effect of the sphere and to compare with the infrared thermometer measurements. The contact probes were Pt100 type and the electronic transductors were calibrated with a resistance box provided by CEM. Finally, the sphere and the infrared thermometer were placed in the field, in an electric substation sited in the north of Spain, in collaboration with the electric distribution company, Viesgo, as can be seen in Figure 7.

## 3. Results and Discussion

In this section, temperature values of the measurements done by the Pt100 near the sphere, $t_{c}$, and the infrared thermometer on the sphere surface, $t_{sir}$ are compared and discussed. Measurements were done during almost a year, from August 2016 to April 2017, storing around 150,000 measures of the Pt100 and the infrared thermometer for many different weather and load conditions. Table 4 shows the ranges of the values for the main influential parameters, infrared thermometer temperature, $t_{sir}$, Pt100 temperature, $t_{c}$, solar radiation, *R*, current, *I*, ambient temperature, $t_{a}$, relative humidity, RH, and perpendicular wind speed, *V*.

**Figure 8:**
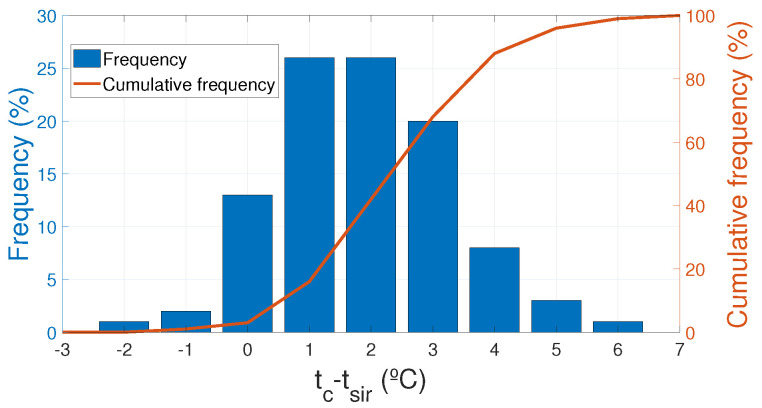
Differences between the Pt100 and the pyrometer.

With all these available data, a statistical approach was done to check the temperature deviation between the infrared thermometer and the contact Pt100 measurements. Figure 8 represents the percentage of the deviation between the Pt100 and the pyrometer and the cumulative deviation of this difference and Table 5 summarizes the main values represented in the Figure 8.

**Figure 9:**
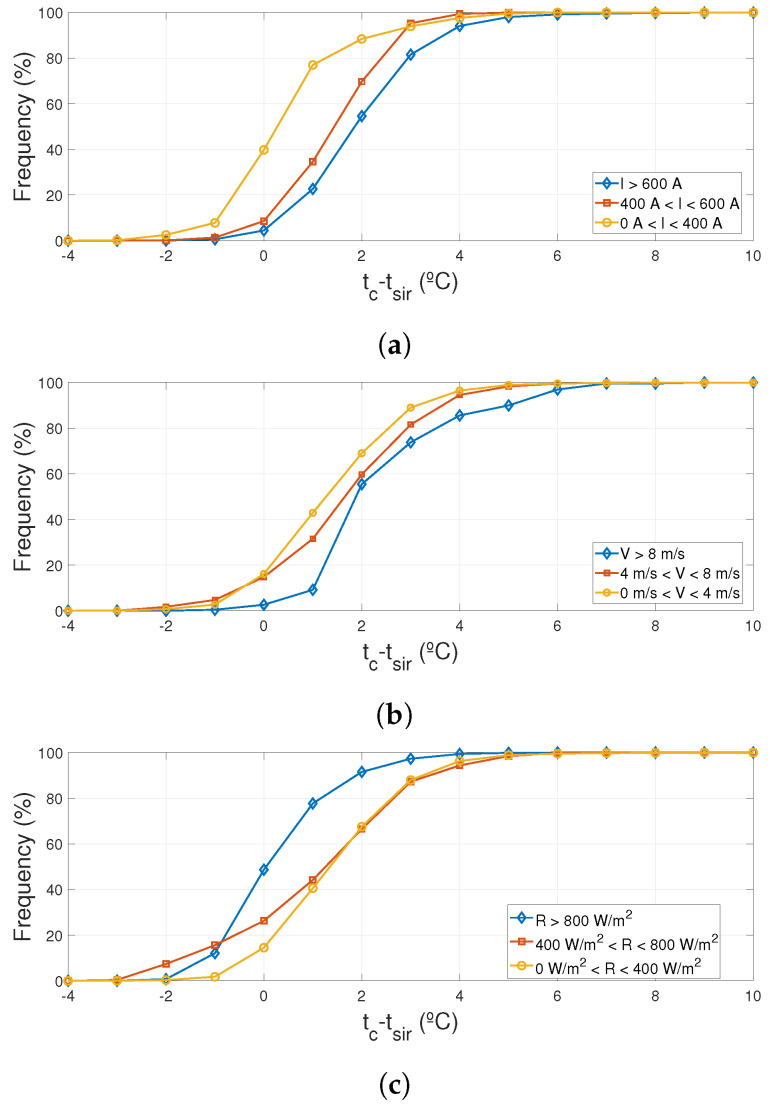
Cumulative frequency of the differences between the Pt100 and the pyrometer. (**a**) Differences between the Pt100 and the pyrometer for different current intervals; (**b**) Differences between the Pt100 and the pyrometer for different wind speeds intervals; (**c**) Differences between the Pt100 and the pyrometer for different solar radiation intervals.

If data are divided by ranges of values of the main influence variables, current, *I*, wind speed, *V*, and solar radiation *R*, several conclusions can be made. As shown in Figure 9a, as the current through the conductor increases the average deviation also increases. The same effect can be seen in Figure 9b with the increase of the wind speed. These results are predicted by the heat transfer theory, as the Joule effect or convection losses increase, the effect of the sphere acting as a fin also increases. Figure 9c represents the variation with solar radiation which tends to homogenize the temperature of the conductor and the sphere as the radiation increases.

Table 6 summarizes the average and standard deviation of the differences between the Pt100 and the pyrometer separated by variable ranges and the total values. The results of 152,000 measurements over a 9-month period (samples taken every 2 min) with a Pt100 contact probe and an infrared thermometer show that the average difference between both measures is $2.39{\;}^{\circ }C$ with a standard deviation of $1.42{\;}^{\circ }C$. Due to the security margins in which the overhead conductors are operated these differences are small enough to be assumable for sag control and ampacity evaluation. The cooling effect of the sphere and a correction function to estimate the conductor temperature far away from the sphere were evaluated in detail in previous studies [12].

## 4. Conclusions

An infrared thermometer was tested to measure the temperature of overhead conductors avoiding the complexity of contact instrumentation in high-voltage lines. A prototype line was built and a system corresponding with the infrared thermometer and a sphere attached to the conductor to increase the spot size was mounted on it. Additionally, a contact temperature measurement with a Pt100 probe was also attached to the conductor and both measurements were compared.

These results conclude that this infrared system (pyrometer and calibrated sphere) can be successfully used to control the temperature of the overhead conductor within a range of less than $4{\;}^{\circ }C$ difference respect contact temperature methods for the 88% of the samples and less than $6{\;}^{\circ }C$ for the 99% with and average difference of $2.39{\;}^{\circ }C$ and a standard deviation of $1.42{\;}^{\circ }C$.

## 5. Patents

A non-contact temperature measurement method has been patented Patent No. ES2542043A122 [11] resulting from the work reported in this paper.

## Figures and Tables

**Table 1:** Simulated sphere temperature for 3 diameters and different weather and line conditions.

Case	Direct Rad. $\left(W/m^{2}\right)$	Diffuse Rad. $\left(W/m^{2}\right)$	Current (A)	Ambient t ${}^{\circ }C$	Perp. Wind Speed (m/s)	Simulated Sphere t (6 cm Diameter) ${(}^{\circ }C)$	Simulated Sphere t (9 cm Diameter) ${(}^{\circ }C)$	Simulated Sphere t (12 cm Diameter) ${(}^{\circ }C)$
1	931	109	677	20	1.7	41.2	31	28.4
2	896	115	681	22	2.8	41.1	30.5	27.1
3	671	73	687	21	4.4	43.4	27.5	25.5
4	0	0	678	18	0.1	63.5	25.4	19.1

**Table 2:** Contact and radiation temperature measurements at CEM.

Sample	$t_{\mathrm{nominal}}$ ${}^{\circ }C$	$t_{\mathrm{cont}}$ ${}^{\circ }C$	$\sigma ,{\;}^{\circ }C$	$t_{\mathrm{rad}}\left(\epsilon =1\right)$ $C300_{\mathrm{painted}}$ ${}^{\circ }C$	$\sigma ,{\;}^{\circ }C$	$t_{\mathrm{rad}}\left(\epsilon <1\right)$ $C300_{\mathrm{unpainted}}$ ${}^{\circ }C$	$\sigma ,{\;}^{\circ }C$	$\left(t_{\mathrm{cont}}-t_{\mathrm{rad}}\right)$ ϵ=1 ${}^{\circ }C$	$\left(t_{\mathrm{cont}}-t_{\mathrm{rad}}\right)$ ϵ=1 ${}^{\circ }C$	$t_{\mathrm{rad}}\left(\epsilon =1\right)-t_{\mathrm{rad}}$ (ϵ<1) ${}^{\circ }C$	$U{,}^{\circ }C$
	5	3.89	0.06	1.70	0.07	4.10	0.14	2.19	−0.21	−2.40	1.40
New conductor	22	21.84	0.03	20.90	0.04	20.00	0.04	0.94	0.84	−0.10	0.70
	50	49.92	0.03	50.00	0.05	28.70	0.03	−0.08	21.22	21.30	0.50
	80	85.12	0.10	86.70	0.08	39.90	0.05	−1.58	45.22	46.80	1.10
	5	7.55	0.16	5.70	0.10	6.50	0.05	1.85	1.05	−0.80	1.20
Used conductor	22	21.94	0.03	21.10	0.04	21.10	0.04	0.84	0.84	0.00	0.70
	50	47.50	0.13	48.30	0.08	44.30	0.07	−0.80	3.20	4.00	0.70
	80	78.43	0.14	80.90	0.12	72.50	0.07	−2.47	5.93	8.40	1.60
	5	2.47	0.09	0.90	0.07	0.50	0.05	1.57	1.97	0.40	1.10
Sphere	22	21.73	0.03	20.80	0.04	20.80	0.05	0.93	0.93	0.00	0.70
	50	48.55	0.06	52.20	0.09	52.90	0.06	−3.65	−4.35	−0.70	2.20
	80	81.76	0.03	89.20	0.17	91.40	0.06	−7.44	−9.64	−2.20	4.30

**Table 3:** Emissivity values of the pyrometer to obtain the temperature differences measured.

Sample	$t_{\mathrm{nominal}}$ ${}^{\circ }C$	$t_{\mathrm{contact}}$ ${}^{\circ }C$	$t_{\mathrm{rad}}\left(\epsilon =1\right)$ ${}^{\circ }C$ $C300_{\mathrm{painted}}$	$t_{\mathrm{rad}}\left(\epsilon <1\right)$ ${}^{\circ }C$ $C300_{\mathrm{unpainted}}$	Emissivity C300 $t_{\mathrm{cont}}\left(\epsilon =1\right)$	Emissivity C300 $t_{\mathrm{rad}}\left(\epsilon =1\right)$
	5	3.89	1.70	4.10	0.99	0.88
New conductor	22	21.84	20.90	20.00	-	-
	50	49.92	50.00	28.70	0.52	0.52
	80	85.12	86.70	39.90	0.47	0.46
	5	7.55	5.70	6.50	0.93	0.96
Used conductor	22	21.94	21.10	21.10	-	-
	50	47.50	48.30	44.30	0.89	0.86
	80	78.43	80.90	72.50	0.88	0.84
	5	2.47	0.90	0.50	0.90	0.98
Sphere	22	21.73	20.80	20.80	-	-
	50	48.55	52.20	52.90	0.85	0.97
	80	81.76	89.20	91.40	0.82	0.95

**Table 4:** Range of values of the main influential parameters.

	$t_{\mathrm{sir}}$ ${(}^{\circ }C)$	$t_{c}$ ${(}^{\circ }C)$	*R* $\left(w/m^{2}\right)$	*I* (A)	$t_{a}$ ${(}^{\circ }C)$	RH %	*V* (m/s)
Max	71.10	73.28	1274.00	702.20	28.28	100.00	12.56
Min	6.10	7.85	0.00	233.60	2.19	33.43	0.04
Mean	27.32	29.71	100.90	529.23	14.28	78.88	2.30

**Table 5:** Main values of deviation between the Pt100 and the pyrometer.

$\left(t_{c}-t_{\mathrm{sir}}\right){(}^{\circ }C)$	−3.00	−2.00	−1.00	0.00	1.00	2.00	3.00	4.00	5.00	6.00	7.00
Deviation $\left(t_{c}-t_{sir}\right)$ (%)	0.00	1.00	2.00	13.00	26.00	26.00	20.00	8.00	3.00	1.00	0.00
Cumulative deviation $\left(t_{c}-t_{sir}\right)$ (%)	0.00	0.00	1.00	3.00	16.00	42.00	68.00	88.00	96.00	99.00	100.00

**Table 6:** Average and standard deviation of the differences between the Pt100 and the pyrometer.

	Range	Standard Deviation (${}^{\circ }$C)	Average Deviation (${}^{\circ }$C)
**V (m/s)**	0–4	1.40	2.35
	4–8	1.55	1.58
	>8	1.58	3.37
**R (w/m${}^{2}$)**	0–400	1.38	2.43
	400–800	1.86	2.09
	>800	1.18	1.22
**I (A)**	0–400	1.32	1.46
	400–600	1.04	2.40
	>600	1.32	2.95
**Total data**		1.42	2.39
